# Supplementary material for: Upgraded User-Friendly Image-Activated Microfluidic Cell Sorter Using an Optimized and Fast Deep Learning Algorithm
Source: Micromachines (Basel). 2022 Nov 29;13(12):2105. doi: 10.3390/mi13122105 (PMC9783339; doi:10.3390/mi13122105)
Supplement: Supplementary file 1 [file micromachines-13-02105-s001.zip › Supplementary data_micromachines.pdf]

Supplementary data

Upgraded user-friendly image-activated microfluidic cell sorter using an optimized and fast deep learning algorithm

Keondo Lee[1], Seong-Eun Kim[1], Seokho Nam[1], Junsang Doh[2], and Wan Kyun Chung[1*]

[1]Department of Mechanical Engineering, Pohang University of Science and Technology, Pohang 37673, Korea

[2]Department of Materials Science and Engineering, Seoul National University, Seoul 08826, Korea

* Correspondence: wkchung@postech.ac.kr (W.K.C.)

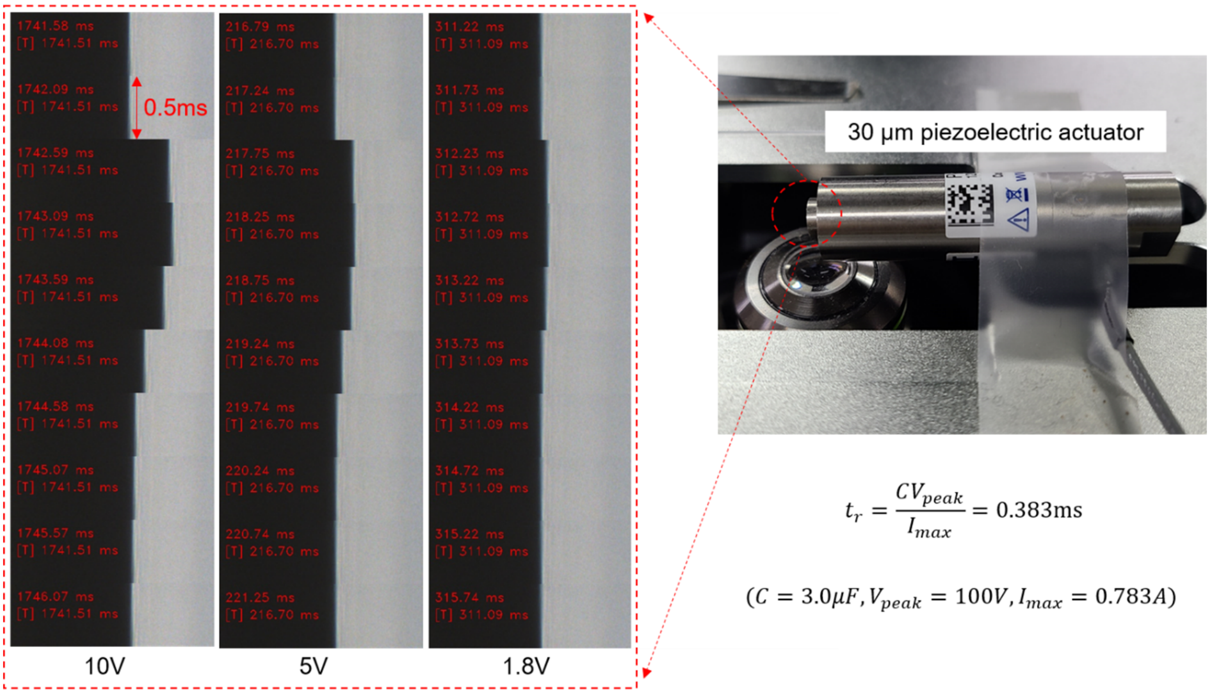

$$t_r = \frac{CV_{peak}}{I_{max}} = 0.383\text{ms}$$

$$(C = 3.0\mu F, V_{peak} = 100V, I_{max} = 0.783A)$$

Figure S1. Microscopic images recorded at 2000 fps show how a 30 μm piezoelectric actuator expands and contracts after a sorting signal. The calculated rising time of the 30 μm piezoelectric actuator is 0.383 ms at 100V.

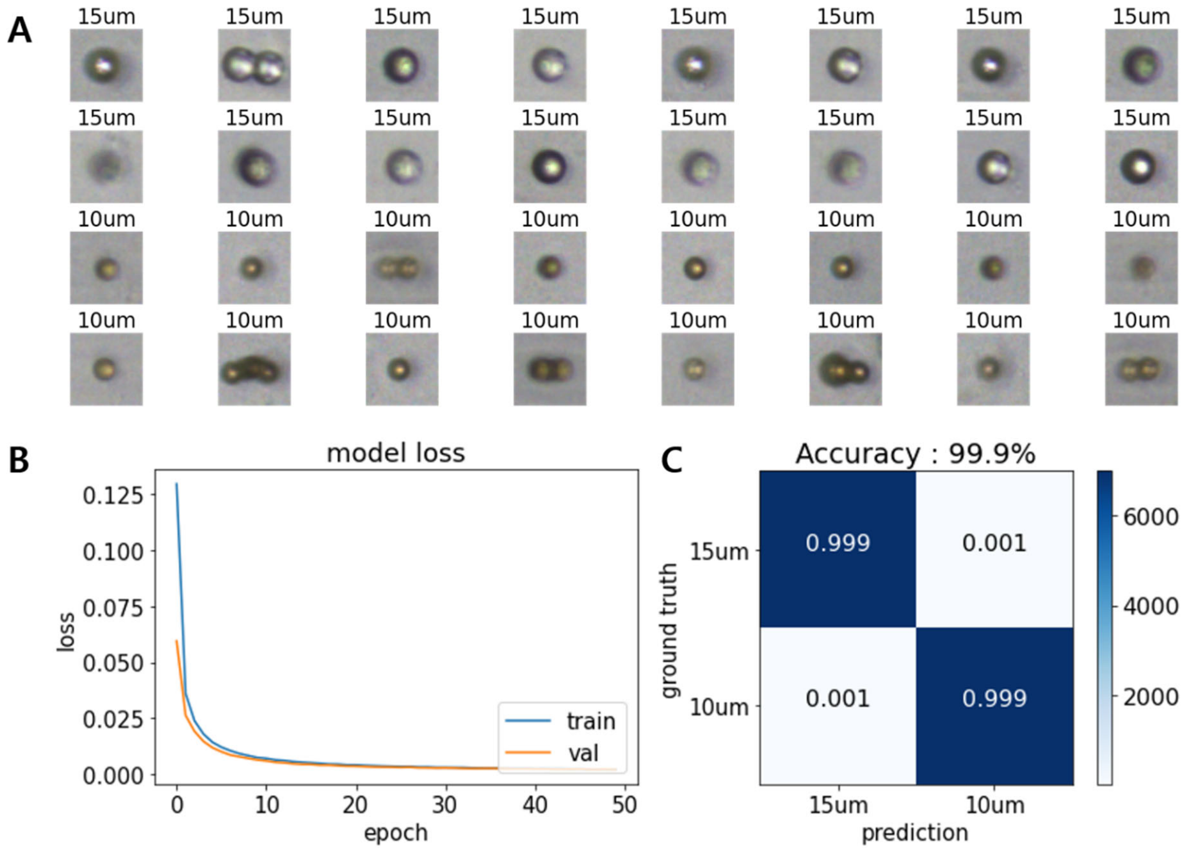

Figure S2. (A) Fluorescent bead images obtained using the upgraded user-friendly image-activated microfluidic cell sorter, (B) Training Learning Curve, (C) Confusion matrix on the test set. 56,000 images of fluorescent beads were trained by ResNet18 for 50 epochs.

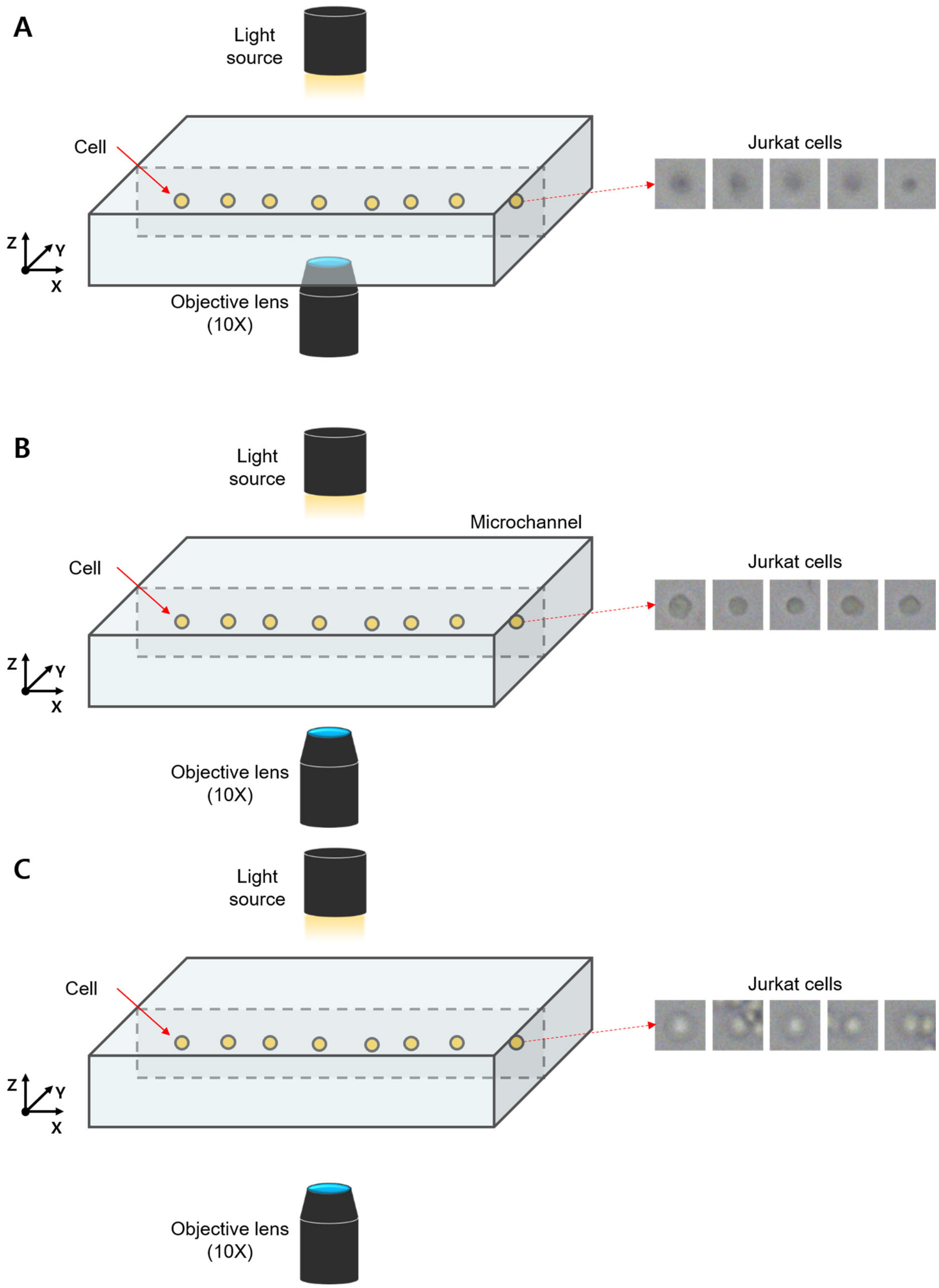

Figure S3. (A) Out of focus (too close), (B) In focus, (C) Out of focus (too far)

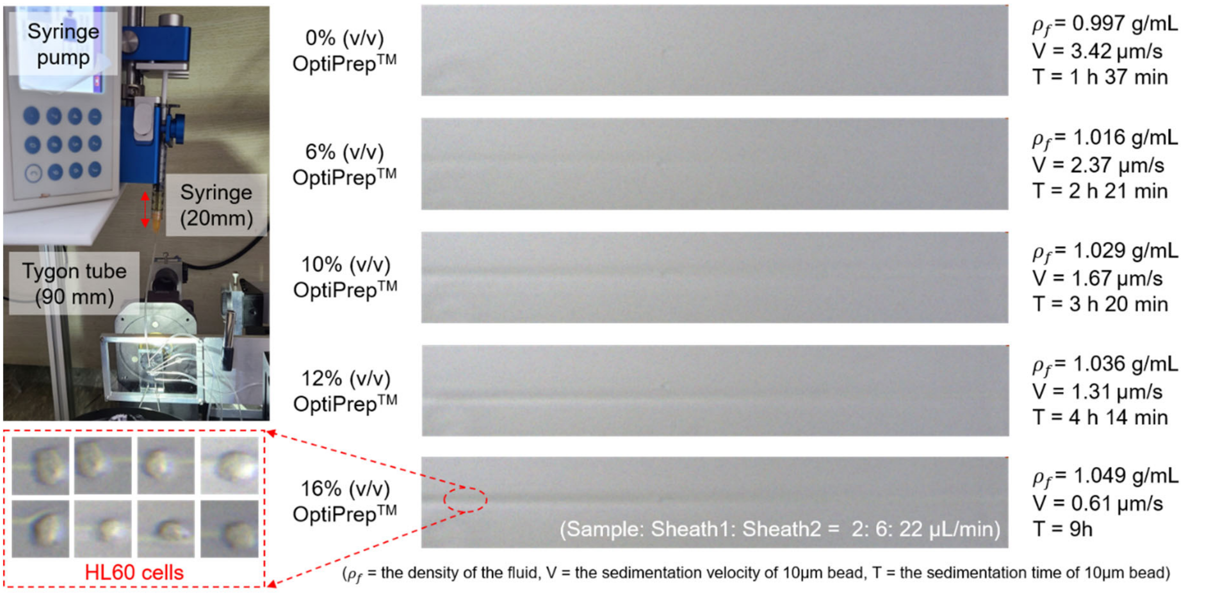

Figure S4. Microscopic images of focused sample flow according to OptiPrep^{TM} concentration. As the OptiPrep^{TM} concentration increases, the focused stream is photographed together in the background of the cell image. When OptiPrep^{TM} concentration is 0%, the 10 μm beads sink into the inlet of the syringe, which takes about an hour and 37 minutes.

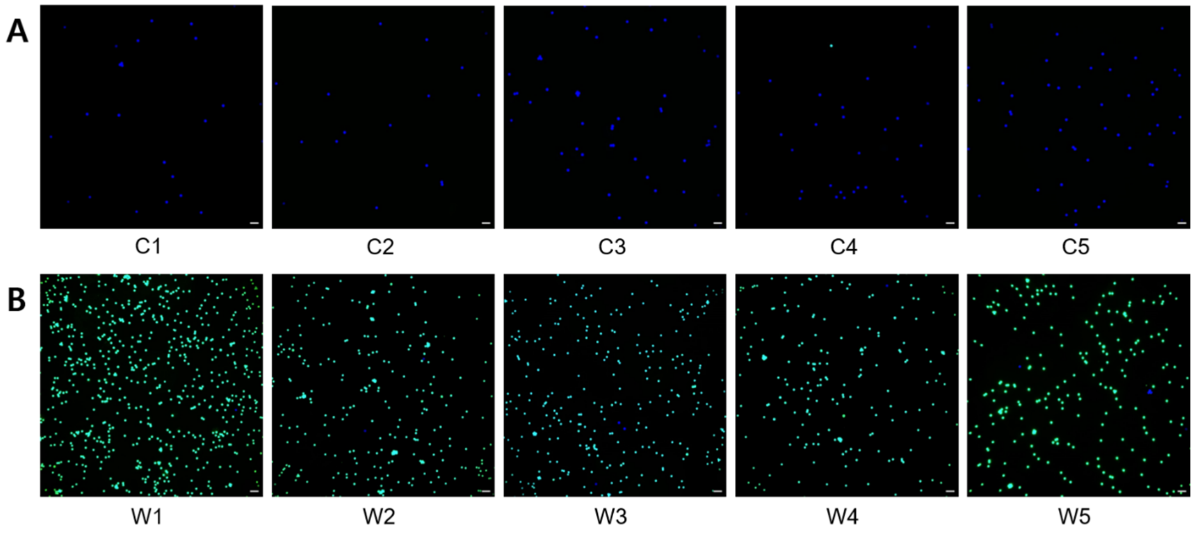

Figure S5. Fluorescence microscopy images of five consecutive bead sorting performed on the upgraded system (scale bar 100 μm). The blue and green dots indicate 15 μm and 10 μm beads, respectively. (A) Collection channel (Post-sorting), (B) Waste channel.

Table S1. Ratios of 15 μm and 10 μm beads in collection and waste outlets. The results in this table were evaluated by manually counting the dots with a fluorescence microscope.

	15μm (Blue)	10μm (Green)		15μm (Blue)	10μm (Green)
C1	99.7%	0.3%	W1	0.2%	99.8%
C2	100.0%	0.0%	W2	0.1%	99.9%
C3	98.5%	1.5%	W3	0.5%	99.5%
C4	99.2%	0.8%	W4	0.5%	99.5%
C5	99.7%	0.3%	W5	2.9%	97.1%
Pre-sorting ratio	14.9%	85.1%			
